# Reassessing the Role of the Neurofilament Light Chain in Guillain‐Barre Syndrome: Issues in Diagnosis and Subgroup Classification

**DOI:** 10.1111/ene.70060

**Published:** 2025-02-05

**Authors:** Hung Youl Seok, Mi‐Yeon Eun

**Affiliations:** ^1^ Department of Neurology, School of Medicine Kyungpook National University, Kyungpook National University Hospital Daegu Republic of Korea; ^2^ Department of Neurology, School of Medicine Kyungpook National University, Kyungpook National University Chilgok Hospital Daegu Republic of Korea

**Keywords:** diagnosis, Guillain‐Barre syndrome, Miller Fisher syndrome, neurofilament light chain

Dear Editor,

We found the paper by Afzali et al. insightful, particularly the demonstration that serum neurofilament light chain (NfL) levels can differentiate acute inflammatory axonal polyneuropathy (AIAP) and predict disease severity [1]. However, there are several issues that need to be addressed before fully accepting the findings.

First, there are issues with the diagnosis and subgroup classification of Guillain‐Barre syndrome (GBS) patients in this study. The authors state that GBS was diagnosed using the National Institute of Neurological Disorders and Stroke (NINDS) and Brighton criteria, with patients classified into subgroups (acute inflammatory demyelinating polyneuropathy [AIDP], AIAP, and Miller Fisher syndrome [MFS]) based on electrodiagnostic criteria by Rajabally et al. [1]. However, both the NINDS and Brighton criteria require symmetrical flaccid weakness for a GBS diagnosis [2]. However, table 1 in the paper by Afzali et al. shows that 15.15% of AIDP patients and 12.5% of AIAP patients have no motor weakness [1]. This raises the question: can these patients really be classified as having classic GBS?

Additionally, although the authors used Rajabally's criteria to classify AIDP, AIAP, and MFS [1], these criteria were designed to differentiate AIDP from axonal GBS, not to classify MFS [3]. Therefore, applying Rajabally's criteria to MFS is methodologically incorrect. MFS diagnosis should follow the NINDS and Brighton criteria, which require a triad of ophthalmoplegia, ataxia, and decreased reflexes without limb weakness [2]. However, table 1 in the paper by Afzali et al. shows that 53.33% of MFS patients have limb weakness [1], suggesting an MFS–GBS overlap syndrome rather than pure MFS. This discrepancy undermines the results regarding NfL in MFS, especially since previous studies show increased NfL levels in both axonal GBS and MFS [4], contrary to this study's findings. Therefore, the MFS subgroup classification needs clarification, and further research on NfL's role in MFS is needed.

Second, the authors did not provide detailed data on nerve conduction studies (NCS), which are critical to understanding neuroaxonal damage in this cohort. Rajabally's classification distinguishes axonal from demyelinating pathology [3], but does not assess the extent of axonal damage. Correlating NCS findings with NfL levels would provide a clearer understanding of the relationship between axonal damage and NfL levels. Given that more than half of the MFS patients may have MFS–GBS overlap syndrome, NCS results should be examined to determine how many meet the criteria for axonal GBS in MFS patients.

Finally, while the study suggests that serum NfL is a useful biomarker for axonal GBS, the authors may inadvertently imply that NfL is only relevant in this disease, with limited applicability in other diseases such as chronic inflammatory demyelinating polyneuropathy (CIDP). However, previous studies show that NfL is an informative biomarker of disease severity and outcome in CIDP [5]. The authors should clarify that these findings should not exclude NfL's value in CIDP or other immune‐mediated neuropathies.

In conclusion, while Afzali et al.'s study highlights the potential of NfL as a biomarker of neuroaxonal damage in axonal GBS, issues of GBS subgroup diagnosis and MFS classification need to be addressed. Further studies are needed to define NfL's role in various neuroimmunological disorders, especially in MFS.

## Author Contributions

**Hung Youl Seok:** conceptualization, investigation, formal analysis, writing – original draft. **Mi‐Yeon Eun:** conceptualization, supervision, formal analysis, writing – review and editing.

## Ethics Statement

The authors have nothing to report.

## Conflicts of Interest

The authors declare no conflicts of interest.

## Data Availability

Data sharing is not applicable to this article, as no new data were created or analyzed in this study.
